# A Rapid and Simple Method for Microscopy-Based Stomata Analyses

**DOI:** 10.1371/journal.pone.0164576

**Published:** 2016-10-12

**Authors:** Jochen F. Eisele, Florian Fäßler, Patrick F. Bürgel, Christina Chaban

**Affiliations:** Department of Plant Physiology, Center for Plant Molecular Biology (ZMBP), University of Tübingen, Tübingen, Germany; University of Delhi—South Campus, INDIA

## Abstract

There are two major methodical approaches with which changes of status in stomatal pores are addressed: indirectly by measurement of leaf transpiration, and directly by measurement of stomatal apertures. Application of the former method requires special equipment, whereas microscopic images are utilized for the direct measurements. Due to obscure visualization of cell boundaries in intact leaves, a certain degree of invasive leaf manipulation is often required. Our aim was to develop a protocol based on the minimization of leaf manipulation and the reduction of analysis completion time, while still producing consistent results. We applied rhodamine 6G staining of *Arabidopsis thaliana* leaves for stomata visualization, which greatly simplifies the measurement of stomatal apertures. By using this staining protocol, we successfully conducted analyses of stomatal responses in *Arabidopsis* leaves to both closure and opening stimuli. We performed long-term monitoring of living stomata and were able to document the same leaf before and after treatment. Moreover, we developed a protocol for rapid-fixation of epidermal peels, which enables high throughput data analysis. The described method allows analysis of stomatal apertures with minimal leaf manipulation and usage of the same leaf for sequential measurements, and will facilitate the analysis of several lines in parallel.

## Introduction

The opening and closing of stomata is a fine-controlled masterpiece of plant evolution driven by the transition of a chemical signal into a mechanical movement. By changing the osmotic pressure in the guard cells, these tiny pores regulate leaf temperature, water evaporation and gas exchange, processes essential for plant survival and growth [1–3]. Due to uptake of CO_2_, stomata participate in providing a carbon source for photosynthetic reactions, whereas the coinciding transpiration of water is essential for nutrient uptake from soil to the plant body. On the other hand, excess water loss from plants under drought stress is disadvantageous and might exert damaging effects resulting in plant death. Because of the great importance of proper stomatal movement, numerous signaling systems inside the plant co-participate in the regulation of stomatal opening and stomatal closure. As a consequence, a mutation of individual components of any of these systems often modulates stomatal movement, which can be either beneficial or unfavorable for the plant. Therefore, the monitoring of stomatal behavior is very important for plant scientists.

Stomatal aperture is tightly regulated by divergent exogenous stimuli, such as light, drought stress, pathogens, temperature and others. These stimuli are sensed and signaled to the guard cells via endogenous signaling molecules including phytohormones, hydrogen peroxide (H_2_O_2_) and Ca^2+^ ions [1,2]. Abscisic acid (ABA) is among the major players in terms of stress related stomatal closure [4]. Elevated ABA concentrations induce multiple cascades of biochemical events like protein phosphorylation, generation of nitric oxide (NO) and H_2_O_2_, changes in intracellular Ca^2+^ concentration and membrane depolarization [1,5–7]. These signaling cascades lead to modifications in the activity of ion channels, decrease of the osmotic pressure in guard cells and, thereby, closure of stomata [1,2,8].

Other phytohormones, such as ethylene, jasmonates and salicylic acid, also function in the regulation of stomatal aperture. Signaling pathways triggered by hormones, as well as by pathogen attack, often involve the generation of second messengers like NO and H_2_O_2_. Treatment of plants with exogenous H_2_O_2_ alone can trigger stomatal closure [6,7]. One pathway by which H_2_O_2_, derived from endogenous and environmental stimuli, is sensed and transduced to effect stomatal closure involves histidine kinase AHK5 [9]. *Arabidopsis* mutants lacking AHK5 have been shown to exhibit impaired stomatal closure in response to H_2_O_2_. Abiotic or hormone signals able to generate endogenous H_2_O_2_, such as darkness or ethylene, also caused reduced stomatal closure in the *ahk5* mutants, whereas stomatal movement was rescued by over-expression of AHK5. An integral signaling function for AHK5 in H_2_O_2_-induced stomatal closure is, however, independent of ABA signaling [9].

Auxin is known to be a positive regulator of stomatal opening although it can also inhibit stomatal opening when applied exogenously at high concentrations [1,2]. Auxin promotes the activation of plasma membrane localized H^+^-ATPases which leads to a hyperpolarization of the membrane. The resulting activation of the K^+^-channels mediates an influx of potassium ions followed by opening of the stomata [1,10].

In spite of the critical role played by stomata in the regulation of plant gas exchange and water use efficiency, the measurement of stomatal aperture is difficult and depends on various environmental factors. Therefore, it is important to have a fast and reliable method to properly monitor stomatal aperture. Here we present a new staining method for stomata, which enables imaging of stomata within minutes after its application. It allows the *in situ* capture of stomata without removing them from their natural surroundings. Additionally, it provides a possibility to analyze stomatal density and stomatal index owing to a staining of pavement cells. As the staining of the stomata takes only 1–2 minutes, one may create snapshots of stomata at short time intervals. Especially, almost instant cell fixation, when desirable, makes it possible to analyze a great number of stomata without the risk of artefact creation due to the prolonged time necessary for the microscopic analyses.

## Materials and Methods

### Plant Material and Growth Conditions

*Arabidopsis thaliana* (L.) Heynh seeds were sown on soil and grown under short day conditions (8 h light/ 16 h dark, photosynthetically active radiation (PAR) of 100–120 μmol m^-2^ s^-1^) at 21°C and 50% relative humidity in controlled environment of growth chambers. Four to six week old plants were used for the analyses. The ecotypes Columbia (Col-0) and Wassilewskija (Ws-4) as well as lines with altered AHK5 expression levels were used for the analyses. The T-DNA insertion line *ahk5-1* is in Col-0 background, *ahk5-3* T-DNA insertion line is in Ws-4 background; P_35S_::GFP:AHK5 is overexpressed in *ahk5-1* background (further referred to as AHK5ox). The lines are described in more detail in Desikan et al. [9]. The *ahk5-1* seeds were originally obtained from Syngenta (SAIL collection), and the *ahk5-3* seeds from the INRA/FLAG-FST collection at Versailles [9].

### Solutions and Treatment

To examine the effect of different signals on stomatal movement, leaves were detached from plants and floated with the abaxial side turned down in a Petri dish containing MES/KOH buffer (5 mM KCl, 10 mM MES (2-(N-morpholino)ethanesulfonic acid), 50 μM CaCl_2_, pH 6.15;[9]). For the treatments, 10 μM ABA (Duchefa), 5 μM IAA (Duchefa) or 100 μM H_2_O_2_ (Sigma) in MES/KOH buffer were used. Unless stated otherwise, all treatments were performed under constant light conditions (combination of Lumilux 18W/840 cool white and L18W/77 fluora lamps from Osram, 100–120 μmol m^-2^ s^-1^ PAR).

For cell staining, rhodamine 6G (Sigma) at final concentration of 0.5–1 μM (dissolved in water) was used. It was freshly prepared from 1mM stock solution (in water), which was kept in darkness. When indicated, 4% formaldehyde was added to staining solution. Either whole leaves or epidermal peels were dipped into the staining solution for 1–2 minutes. When desired, staining was followed by short rinsing in corresponding incubation solution.

### Stomata Assays

#### A. Treatment of transgenic AHK5 lines

**Fig 1:**
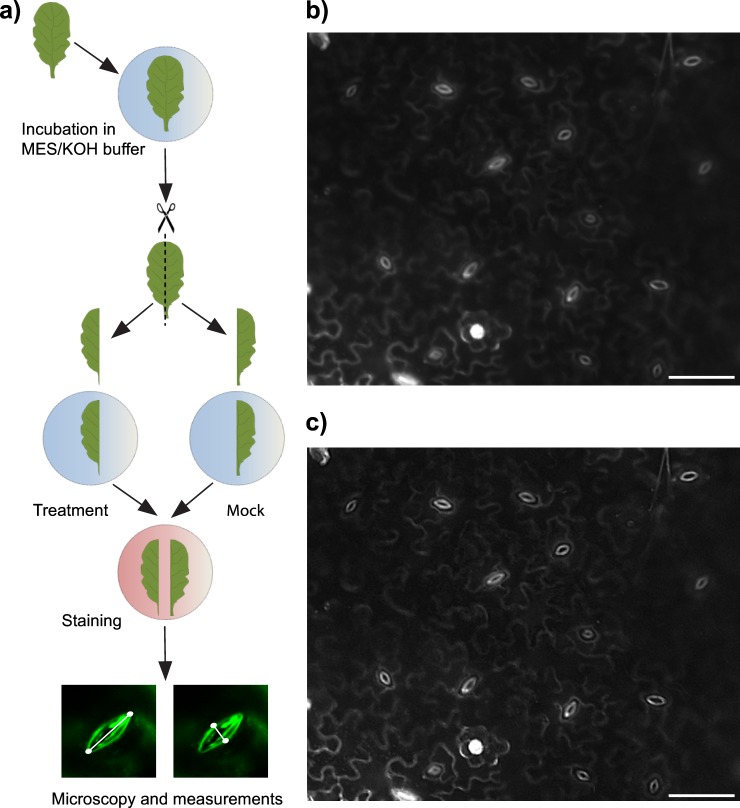
Experimental setup for stomatal aperture measurements. (a) Schematic representation of the workflow; (b) epifluorescent microscopic picture of the *Arabidopsis* leaf stained with rhodamine 6G; (c) the same picture as in (b) after application of the option “sharpen” in ImageJ. Bars, 50 μm.

Three fully expanded leaves at a comparable developmental stage (one leaf per plant) were used per line for each treatment. After being detached, leaves were incubated in the MES/KOH buffer in Petri dishes for 2 hours for equilibration and stomata opening [9]. Afterwards the leaves were cut along the midrib. One part of the leaf was further incubated in MES/KOH buffer for 2 h (mock-treated control), whereas the other part was treated either with ABA or with H_2_O_2_ for 2 h (Fig 1A). After treatment, both leaf parts were stained as described above and examined under microscope. The data are presented in comparison to the corresponding controls (mock treatments).

#### B. Phytohormone treatment (intact leaves)

Three leaves per treatment (one leaf per plant at a comparable developmental stage) were incubated in MES/KOH buffer for 2 h. Then the leaves were cut along the midrib and treated as follows: one leaf half was stained in the staining solution and immediately examined under microscope (corresponds to 0 h treatment); then it was incubated either in ABA or IAA for 2 h followed by examination under microscope (corresponds to 2 h treatment). Another leaf half was incubated either in ABA or IAA for 2 h without pre-staining, then stained in the staining solution and examined under microscope (corresponds to 2 h post-stained).

For measurements of stomatal closure kinetics, after pre-incubation in MES/KOH buffer, three leaves per treatment and time point (i.e. 0 h, 30 min and 1 h), hence a total of nine leaves, were cut along the midrib and treated as follows: half of all leaves was pre-stained, then transferred to ABA-containing MES/KOH buffer for different time periods; the other half was transferred to ABA-containing MES/KOH buffer without pre-staining and stained immediately before microscopic analysis (post-stained). The stomatal aperture for 0 h time point was measured before ABA treatment.

#### C. Phytohormone treatment (epidermis peels)

Epidermis peels were prepared as described in Wu et al. [11] with small modifications. The adaxial epidermal leaf surface was affixed to a strip of laboratory tape (TimeMed Labeling Systems, Inc.) with the abaxial side facing upwards. A strip of transparent universal adhesive tesa film (solvent free) (Tesa SE) was gently applied to the abaxial surface of the affixed leaf. The tesa adhesive tape was then carefully pulled away from the laboratory tape, peeling away the abaxial epidermal cell layer. Adhesive tape with attached epidermis peels was immediately transferred to a Petri dish containing MES/KOH buffer and incubated for 2 h. Then the peels were transferred either to MES/KOH buffer (control / mock) or to ABA-containing MES/KOH buffer for 2 h. At the end of the incubation period, the epidermal cell peels were stained and analyzed under microscope. To enable a direct comparison, the intact leaves were treated in parallel with the peels. For the stomatal aperture closure kinetics in response to ABA, the images of guard cells were taken under microscope at 0 h, 0.5 h and 1 h.

#### D. Experiment using cell fixation

Epidermis peels were prepared from twelve leaves at a similar developmental stage (one leaf per plant) and put in Petri dishes containing MES/KOH buffer. The Petri dishes were maintained either under light or in darkness for 2 h. Then one part of the peels was immersed for 30 s into staining solution containing 4% formaldehyde and returned to MES/KOH buffer, whereas the other part was pre-stained in the formaldehyde-free solution. The peels were imaged employing an epi-fluorescence microscope (Nikon Eclipse 90i) and returned to the corresponding petri dishes. The petri dishes previously maintained under light were transferred into darkness, whereas those from darkness were transferred into light conditions for 30 min. At the end of the treatment the peels were again analyzed under microscope. Peels from three leaves per treatment were used for analysis.

### Measurement and Analysis of Stomatal Aperture

After staining, imaging of stomata was performed with a Nikon Eclipse 90i microscope equipped with a CCD camera using a TRITC filter Ex 540/25 DM 565 BA 605/55 (Nikon). Image analysis was performed using ImageJ software (https://imagej.nih.gov/ij/). For better visualization of the guard cells the option “sharpen” in ImageJ was used. The width and the length of the stomatal aperture were measured as shown on Fig 1A, and the stomatal aperture index (SAI) was calculated by division of the aperture width through the length. The SAI of at least 30 stomata per leaf was calculated, and three leaves per each treatment / time point were used for statistical analysis. Data were analyzed using Student’s t-test. The confocal images were captured under Leica SP8 microscope at following settings: excitation at 488 nm, emission 505–545 nm (green fluorescence); excitation at 561 nm, emission at 600–640 nm (red fluorescence), 20x objective, using Leica Application Suite (LAS) software.

## Results

### Stomatal Closure Analysis in Transgenic Lines with Altered AHK5 Levels

Rhodamine 6G belongs to a group of fluorescent dyes widely used in different fields including molecular and cell biology. Different rhodamine derivatives are most often applied as fluorescent reporters fused to antibodies or other molecules in order to visualize their intracellular localization. Rhodamine 6G is characterized by a high quantum yield (0.90–0.98%) and its fluorescence can be detected even at concentrations as low as 10^−7^ g/ml [12,13]. From preliminary experiments we found that the optimal staining of guard cells was achieved at 0.1–1 μM rhodamine 6G. At this concentration the guard cells are often more strongly stained than the pavement cells (Fig 1B). The fluorescent stomata can be visualized either with green (emission wavelength λ~520 nm), yellow-orange (emission wavelength λ~530–560 nm) or red (emission wavelength λ~600 nm) filters. Using the option “sharpen” in ImageJ allows a better recognition of stomatal boundaries (Fig 1C).

**Fig 2:**
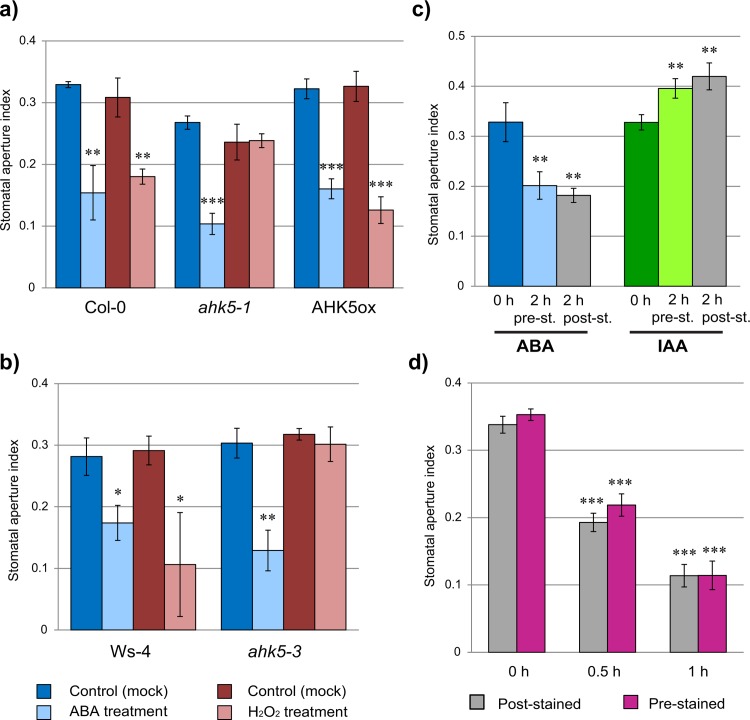
Changes in stomatal apertures in *Arabidopsis* leaves. Stomata closure in response to 10 μM ABA and 100 μM H_2_O_2_ in Col-0, *ahk5-1* and AHK5 overexpressor lines (a) and Ws-4 and *ahk5-3* mutant line (b). Three fully expanded leaves at a similar developmental stage (one leaf per plant) per treatment were analyzed for each line. A single asterisk indicates a significant difference to corresponding mock-treated leaves (*, 0.01 < p ≤ 0.05), two asterisks depict a very significant difference (**, 0.001 < p ≤ 0.01), and three asterisks indicate an extremely significant difference to corresponding mock-treated leaves (***, p ≤ 0.001). (c,d) Comparison of stomatal movements in rhodamine 6G pre-stained (before treatment with hormones) and post-stained *Arabidopsis* leaves. (c) Stomatal apertures in leaves treated with 10 μM ABA or 5 μM IAA for 2 h. *pre-st*.: pre-stained leaves, *post-st*.: post-stained leaves. (d) Stomatal apertures in leaves treated with 10 μM ABA for 30 min and 1 h. A total of six leaves (c) and nine leaves (d) at a similar developmental stage (one leaf per plant) were used for the analyses. A very significant difference between leaves before treatment (0 h) and after treatment is indicated by two asterisks (**, 0.001 < p ≤ 0.01), an extremely significant difference is indicated by three asterisks (***, p ≤ 0.001).

Although very low dye concentrations were used for staining, there was still the chance of rhodamine 6G affecting stomatal aperture. Therefore, we analyzed stomatal apertures in *Arabidopsis* lines with altered levels of AHK5 and corresponding background ecotypes in response to ABA and H_2_O_2_. It had been previously shown that AHK5 functions in promoting H_2_O_2_-dependent, but not ABA-dependent stomatal closure [9]. Two lines with T-DNA insertion in *AHK5* gene, *ahk5-1* in Col-0 and *ahk5-3* in Ws-4 background, as well as AHK5 overexpressor line in *ahk5-1* background (AHK5ox) [9] were used in the analysis. Both wild-type *Arabidopsis* ecotypes reacted to ABA and H_2_O_2_ with stomatal closure (Fig 2A and 2B). In consistence with previously published results, *ahk5* mutant lines failed to close stomata in response to H_2_O_2,_ irrespective of the ecotype background. Moreover, ectopic expression of AHK5 in *ahk5-1* background led to the reversion of the mutant phenotype, i.e. stomatal apertures were comparable with those in wild-type leaves. With respect to ABA, mutant lines still reacted similarly to wild type demonstrating stomatal closure (Fig 2A and 2B). The obtained data indicate that applied rapid staining of leaves immediately before analysis allows visualization of stomatal pores on intact leaves and proper evaluation of stomatal aperture without detectable side effects on stomatal behavior.

### Analyzing the Long-Term Effect of Rhodamine Staining on Stomatal Movement

Our next question was whether rhodamine influences stomatal movement in pre-stained leaves. Therefore, after detachment, the leaves of Col-0 plants were either stained with rhodamine and then incubated in ABA- or IAA-containing buffer, or incubated with phytohormones without rhodamine pre-staining. In the latter case, the leaves were stained immediately before image acquisition (further referred as to post-stained). Incubation of leaves in 10 μM ABA induced stomatal closure in both pre-stained and post-stained leaves (Fig 2C). The stomatal aperture after 2 h treatment was comparable in pre-stained and “post-stained” leaves. Auxin treatment caused further opening of pores compared to untreated control (0 h IAA). The increase of aperture was small yet significant. As in the case of ABA, we did not detect any difference between pre-stained and post-stained stomatal apertures (Fig 2C).

In order to see if the response kinetics of stomata is not impeded by the staining, the monitoring of stomatal closure at several time points in response to ABA was conducted. The decrease of stomatal aperture was already detectable after 30 min of the ABA treatment irrespective of rhodamine pre-staining. The kinetics of stomatal closure was similar in the pre-stained and post-stained leaves (Fig 2D). In conclusion, pre-staining of leaves with rhodamine 6G influenced neither stomatal closure nor opening.

### Stomatal Closure in the Peeled Epidermis

**Fig 3:**
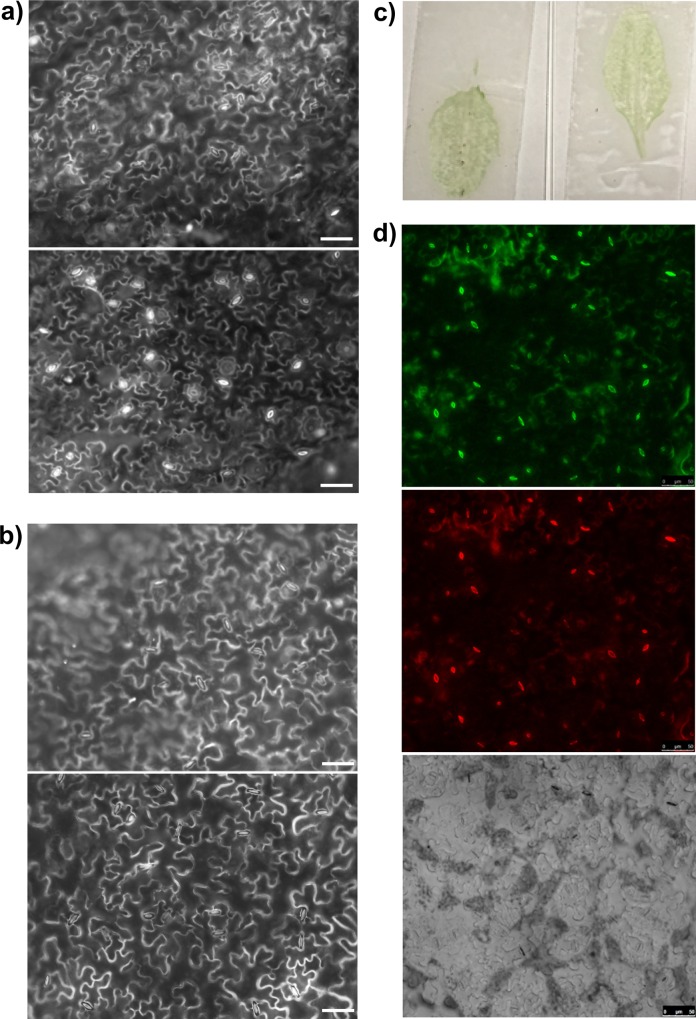
Visualization of stomatal apertures in intact leaves and epidermis peels. (a,b) Epifluorescent images of intact leaves mounted in water (upper panel) and in 30% glycerol (lower panel). (a) Staining with 1 μM rhodamine 6G; (b) staining with 10 μM rhodamine 6G. (c) Photograph of leaf epidermis peels. (d) Confocal image of cells in the peeled epidermis; λ_exc_ = 488 nm, λ_em_ = 505–545 nm (upper panel); λ_exc_ = 561 nm, λ_em_ = 600–640 nm (middle panel); bright field (lower panel). Bars, 50 μm.

As mentioned above, low-concentration rhodamine 6G staining of intact leaves often results in the preferential staining of guard cells as compared to pavement cells. Such differential staining is especially observed in leaves with open stomata. Although this can be advantageous for stomatal aperture measurement, it causes difficulties in analyzing stomatal density or stomatal index. By increasing the dye concentration to 1–10 μM, staining of all epidermal cells is mostly achieved, although it is difficult to acquire images with all cells within the focal plane (Fig 3A and 3B, upper panel). The use of 30% glycerol as an embedding medium for slide preparation significantly improved the acquisition of such images (Fig 3A and 3B, lower panel). Thus, rhodamine-stained epidermis peels can be used for calculating stomatal index and stomatal density.

In the case of stomatal aperture measurement, however, the complications with focusing on numerous cells still persist, since the mounting of leaves in glycerol solution would affect stomata aperture. This results in a considerable prolongation of the microscopic analysis. In order to overcome this problem, leaf epidermis peels are often used [14–16]. To prepare the epidermis peels we applied a very easy method previously used for protoplast isolation [11]. By this method, a peel of broad leaf surface can be readily obtained (Fig 3C). Due to the leveled arrangement of cells in such peels, staining them with rhodamine 6G results in a simultaneous visualization of guard cells throughout the entire section of a sample (Fig 3D).

**Fig 4:**
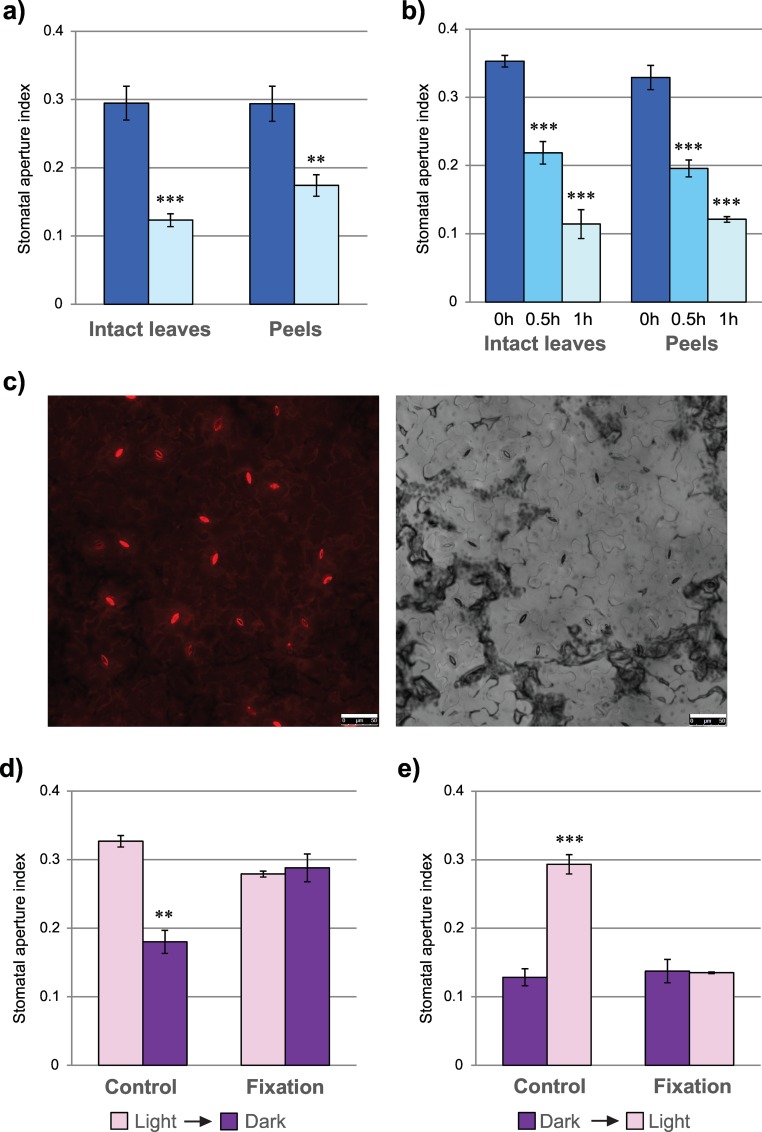
Analysis of the leaf peeling and rapid fixation method for studies on stomatal responses. (a) Stomatal closure in response to 2 h ABA treatment in intact leaves and epidermis peels. Dark-blue rectangles, mock; light-blue rectangles, 10 μM ABA. (b) Kinetics of stomatal closure in response to ABA in intact leaves and epidermis peels. A total of twelve leaves (one leaf per plant at a comparable developmental stage) have been used in each analysis. Two asterisks indicate a very significant difference to corresponding mock-treated cells (**, 0.001 < p ≤ 0.01), while three asterisks depict extremely significant difference to corresponding mock-treated cells (***, p ≤ 0.001). (c) Confocal images of cells in the peeled epidermis after quick fixation in 4% formaldehyde captured in red and bright field. Bar, 50 μm. (d,e) Comparison of stomatal apertures in fixed and non-fixed epidermis peels. (d) After 2 h pre-incubation in light the peels were analyzed under microscope, transferred into darkness for 30 min. and again analyzed. (e) The peels were pre-incubated in darkness, analyzed under microscope, transferred to light for 30 min. and analyzed again. Twelve leaves at a comparable developmental stage were used for peel preparations and subsequent analyses. Two asterisks indicate a very significant difference (**, 0.001 < p ≤ 0.01), while three asterisks depict extremely significant difference between the results of two subsequent measurements (***, p ≤ 0.001).

Our next question was whether such peels could be used for the analysis of stomatal movement. Therefore, we compared ABA-induced stomatal closure in intact leaves and epidermal peels. After pre-incubation in MES/KOH buffer, plant material was stained in rhodamine 6G and incubated either in 10 μM ABA or in MES/KOH buffer alone. As shown on the Fig 4A, upon 2 h of ABA treatment stomatal aperture significantly decreased in both intact leaves and epidermis peels. Measurement of the kinetics of ABA-induced stomatal closure showed that it was comparable in both material samples (Fig 4B). Therefore, by the application of this peeling method the guard cells remain fully functional and can be used for the analyses of stomatal movement.

### Nearly Instant Cell Fixation Is Achievable in the Case of Epidermis Peels

Finally, we were interested in providing the option of cell fixation, which would be especially important for large-scale analyses. In the epidermis peels, cells are located in one layer and are unprotected by cuticle (cuticle faces adhesive film), so theoretically one-minute incubation in the fixative-containing solution should be sufficient for the penetration of fixative molecules throughout the cells. To check if this holds true, we added 4% formaldehyde to rhodamine solution and proceeded with staining as usual. As a control, staining of peels in formaldehyde-free solution was performed. The responses of stomata to light / dark conditions were analyzed. The fixation of epidermis peels did not influence cell staining (Fig 4C), but totally changed stomatal behavior. When light-adapted peels were transferred to darkness, stomatal aperture significantly decreased in control epidermis, but did not change when the peels were immersed in formaldehyde-containing solution before the transfer (Fig 4D). We also analyzed light-induced stomatal opening. In this case, the epidermal peels floating in MES/KOH buffer were initially kept for 2 h in darkness, then stained either in formaldehyde-containing or -free rhodamine solution and incubated for 2 h under light conditions. Similarly to the previous results, stomatal aperture remained unchanged when peels were pre-fixed, whereas stomatal opening was observed in non-fixed epidermis (Fig 4E). These data clearly demonstrate that dipping of epidermal peels for 3 s into formaldehyde-containing staining solution is sufficient for the fixation of guard cells and the prevention of their responses to subsequent signals.

## Discussion

Stomatal analyses are crucial not only for studying guard cell responses, but for understanding a broad range of issues in plant physiology. Different protocols characterized by various degree of the complexity and time consumption are being used in order to perform such analyses. Here we describe a set of procedures which greatly simplify the measurement of stomatal aperture, index and density. These procedures offer two possibilities, which can be applied depending on the requirements of a particular experiment.

Firstly, stomatal movement can be recorded in the intact leaves resulting in a minimization of leaf wounding, maintenance of the natural extracellular environment and a reduction of leaf manipulation. Due to the multilayered leaf structure, the visualization of guard cells in untreated leaves is quite obscure. Virlouvet and Fromm reported the procedure of stomatal aperture measuring by monitoring autofluorescence of cell walls under confocal microscope in the leaves fixed for 6 h [17]. Due to high-intensity excitation light of shorter wavelengths used for the autofluorescence induction (458 nm [18]), even rapid pictures taking does not prevent the risk of cell photodamaging in living leaves. In contrast, specific attributes of rhodamine 6G used for cell staining in our experiments, such as the absorption maximum at approximately 530 nm, its high photostability and high quantum yield [13], make it suitable for usage with non-damaging light of lower intensities. Our data demonstrate that rhodamine 6G staining did not interfere with stomatal closure and opening in response to various signals in different *Arabidopsis* ecotypes. The kinetics of ABA-induced closure of pre-stained stomata was comparable with the closure kinetics of unstained stomata. Thus, the advantage of the method does not only lie in the undisruptive leaf handling, but also in the opportunity to observe the response of the same leaf to consecutively applied signals. A long-term tracking of stomatal movements can be achieved by the reduction of the exposure of the living sample to phototoxic excitation irradiance. Recently, Chitrakar and Melotto used propidium iodide staining for stomatal analyses in intact leaves [19]. The excitation wavelength of propidium iodide is similar to that of rhodamine 6G, however its emission maximum is significantly shifted to red region of the spectrum compared to rhodamine. Thus, the analysis of propidium iodide requires red filter settings, whereas the visualization of rhodamine 6G is also possible with green filter settings. Additionally, propidium iodide is much more expensive and more toxic for human health than rhodamine.

Secondly, for cases in which analysis of intact leaves is not required we adopted a simple and reliable procedure for the preparation of epidermis peels, which was previously reported for protoplast isolation [11]. We have verified the functionality of guard cells in such peels, which also are very easy to handle due to their being attached to adhesive film. A review of published methods for stomatal analyses has shown that the utilization of epidermal peels is a preferential sample preparation procedure [15,16,20,21]. The reason obviously lies in the obtaining of images of much better quality compared to intact leaves, with recognizable epidermal cell boundaries. However, the peel preparation itself often requires certain skills and experience. There are several procedures including the blending of leaves in water for 1–2 minutes, collecting epidermal fragments on a 100 μm nylon mesh with subsequent transfer to a microscope slide [15,16], peeling of epidermal strips using forceps [21] or using liquid medical adhesive to attach epidermal cell layer [20]. With these procedures, we were only able to get small pieces of epidermis, whereas with the use of adhesive tape the peels of complete abaxial leaf epidermis are usually obtained.

To address the issue of high-throughput imaging of epidermal cells, we established a protocol for cell fixation. Due to a very rapid fixation procedure, comparatively large amounts of plant samples can be prepared during short time intervals. This enables, for example, a comparison of different lines treated simultaneously, thus providing highly comparative experimental conditions.

Overall, the described method should now greatly simplify and speed up analysis of the regulation of stomatal movement and development in response to various signals and in multiple lines. With some adjustments of staining time and / or rhodamine concentration this method should also be applicable to other plant species.
